# Occipital neural dynamics in cannabis and alcohol use: independent effects of addiction

**DOI:** 10.1038/s41598-021-01493-y

**Published:** 2021-11-15

**Authors:** Brandon J. Lew, Anabel Salimian, Tony W. Wilson

**Affiliations:** 1 Institute for Human Neuroscience, Boys Town National Research Hospital, 378 Bucher Drive, Boys Town, NE 68010 USA; 2 College of Medicine, University of Nebraska Medical Center, Omaha, NE USA

**Keywords:** Addiction, Magnetoencephalography, Neuroscience, Cognitive neuroscience

## Abstract

Alcohol and cannabis use disorder (AUD/CUD) are two of the most common addictive disorders. While studies are beginning to understand the neural changes related to acute and chronic use, few studies have examined the independent effects of AUD and CUD on neural oscillatory activity. We examined 45 adults who reported current use of both cannabis and alcohol. Participants underwent the SCID-V to determine whether they met criteria for AUD and/or CUD. Participants also completed a visual-spatial processing task while undergoing magnetoencephalography (MEG). ANCOVA with a 2 × 2 design was then used to identify the main effects of AUD and CUD on source-level oscillatory activity. Of the 45 adults, 17 met criteria for AUD, and 26 met criteria for CUD. All participants, including comparison groups, reported use of both cannabis and alcohol. Statistical analyses showed a main effect of AUD, such that participants with AUD displayed a blunted occipital alpha (8–16 Hz) response. Post-hoc testing showed this decreased alpha response was related to increased AUD symptoms, above and beyond amount of use. No effects of AUD or CUD were identified in visual theta or gamma activity. In conclusion, AUD was associated with reduced alpha responses and scaled with increasing severity, independent of CUD. These findings indicate that alpha oscillatory activity may play an integral part in networks affected by alcohol addiction.

## Introduction

Roughly 20.3 million people aged 12 and above were diagnosed with a substance use disorder (SUD) in the United States in the year 2018. This included 14.8 million people who had an alcohol use disorder (AUD) and 8.1 million who had an another drug use disorder, most commonly cannabis use disorder with 4.4 million people diagnosed^1^. With the changes in legislation regarding legalization of recreational cannabis use across the United States, the rates of cannabis use can only be expected to escalate, which may lead to greater potential health risks across all ages of users^2–4^. Not only are alcohol and cannabis use common separately, using both is also very prevalent and largely understudied. Subbaraman found that in comparing simultaneous versus separate use of alcohol and cannabis in adults who use both, using the two drugs simultaneously was two times as common as using them on separate occasions. Simultaneous use also depicted a higher frequency of cannabis and alcohol ingestion at higher quantities, leading to an increased risk of damaging consequences and potential for misuse^5^. Other studies place alcohol and marijuana use disorders as being highly comorbid in adolescents, emphasizing the misuse nationwide by all ages^6,7^. In analyzing the National Comorbidity Survey, Agosti discovered that alcohol dependence had one of the strongest associations to cannabis dependence, shedding light on how difficult it can be to study individual use disorders in isolation^6^.

Studies focusing on neural oscillations have also contributed key findings in regards to alcohol and cannabis misuse across all ages. Both acute and chronic cannabis use have been linked with decreased spectral power in the gamma range during tasks that utilize 40 Hz auditory click train paradigms^8,9^. Further, some work has shown duration of use effects, suggesting that the long-term chronic use of cannabis may be critical to the decline in gamma oscillations^9^. Other studies have looked at induced theta oscillations as a biomarker for alcoholism, due to the consistent decrease in power found in alcohol users over multiple studies^10–13^. Studies of binge drinking in adults have revealed reduced alpha peak frequency compared to light drinkers, leading to interest in alpha oscillations in the context of prolonged alcohol use and ultimate transition into an alcohol use disorder^14^. Importantly, visual oscillatory activity is among the most reliable^15,16^ and highly studied oscillations, however such activity remains understudied in substance use disorders.

Previous literature has found deficits in visual processing abilities in adults with alcohol dependence^13,17,18^ and cannabis users^19^, but the altered brain regions and neural dynamics underlying these deficits remain far less understood. In regards to oscillatory activity, visual-spatial processing is known to be associated with both bottom up and top down visual processing networks^20^, including multispectral responses across the occipital cortices^21–24^. For example, visual cortical oscillations in the theta band have been associated with bottom up processing and the initial basic sensory coding and organization of external stimuli^24–27^, while gamma band activity has been linked to the processing and integration of information across different brain regions, as well as the registration of stimulus features^28,29^. Meanwhile, alpha activity has been linked to top down visual processing, including filtering and inhibiting incoming visual input^30–33^. Alpha activity during visual processing has been specifically implicated in heavy alcohol use^34^, although the role of use disorder was not studied. Given the critical role of oscillations in visual processing and cognition, understanding alterations in these neural population responses will contribute key data on the origin of cognitive and psychological changes in the context of substance use disorders.

In summary, despite evidence that visual-spatial processing deficits are associated with alcohol and cannabis use, and that oscillatory activity is broadly affected by alcohol and cannabis use, few studies have examined visual oscillatory activity in the context of alcohol and cannabis use disorders. In the current study, we use MEG to study the impact of use disorder on the neural dynamics serving visual-spatial processing by comparing adults who used both cannabis and alcohol and met criteria for AUD and/or CUD, to adults who used both cannabis and alcohol but did not meet criteria for AUD and/or CUD. We specifically focused on cortical oscillatory activity and aimed to decipher the independent effects of alcohol and cannabis use disorders. We hypothesized that altered alpha oscillatory activity would be associated with alcohol use disorder, while aberrant gamma oscillatory activity would be associated with cannabis use disorder.

## Results

### Participants

All 45 enrollees reported regular use of both cannabis and alcohol. These participants were grouped by use disorder according to their SCID interview diagnosis. Of the 45 enrollees, 17 participants met criteria for AUD and 28 did not. For CUD, 26 of the participants met criteria, while 19 did not meet criteria for CUD. Specifically, the subgroups consisted of 17 people currently meeting criteria for only CUD, eight currently meeting criteria for only AUD, nine meeting criteria for both AUD and CUD, and 11 not meeting criteria for any substance use disorder. The groups contained a mix of individuals who were diagnosed with mild, moderate, or severe use disorder(s), but all were diagnosed based on use within the past 12 months. Other substance use disorders were assessed on the SCID interview, and six participants had additional other substance use disorder diagnoses. The four groups were comparable in age and race (Table 1), but were notably unbalanced on sex and this was accounted for statistically. All groups had an average Beck depression total score under 10, which is considered normal^35^.

**Table 1:** Participant demographics by comparison.

	AUD (n = 17)	Non-AUD (n = 28)	CUD (n = 26)	Non-CUD (n = 19)
Age in years	29.2 (9.22)	30.5 (10.56)	28.50 (9.94)	32.11 (9.93)
Gender (F/M)	2/15	12/16	8/18	6/13
Race (Caucasian/African American/other)	12/2/3	22/3/3	7/0/1	5/2/2
Years of education	15.6 (1.82)	15.1 (1.79)	15.48 (1.78)	14.92 (1.81)
AUDIT total score	12.06 (4.55)	4.75 (2.88)	6.92 (4.53)	8.32 (5.70)
CUDIT total score	10.59 (4.96)	12.50 (5.52)	14.15 (4.43)	8.53 (4.81)
Accuracy (%)	89.66 (8.31)	93.63 (6.31)	93.01 (5.07)	90.92 (9.60)
Reaction time (ms)	515.66 (111.77)	522.61 (74.89)	523.00 (87.49)	515.86 (94.26)

Means (standard deviation) for each group. The full sample is represented in the AUD comparison as well as the CUD comparison.

### Neurobehavioral performance

When examining task performance, the ANCOVA model on reaction time showed no effect of AUD (*F*(1,40) = 0.01; *p* = 0.939) or CUD (*F*(1,40) = 0.28; *p* = 0.600). Similarly, the ANCOVA model on accuracy showed no effect of AUD (*F*(1,40) = 3.73; *p* = 0.060) or CUD (*F*(1,40) = 0.32; *p* = 0.572). Overall, average accuracy of all participants was 92.13% and mean reaction time was 519.99 ms.

### Sensor-level analyses

**Figure 1:**
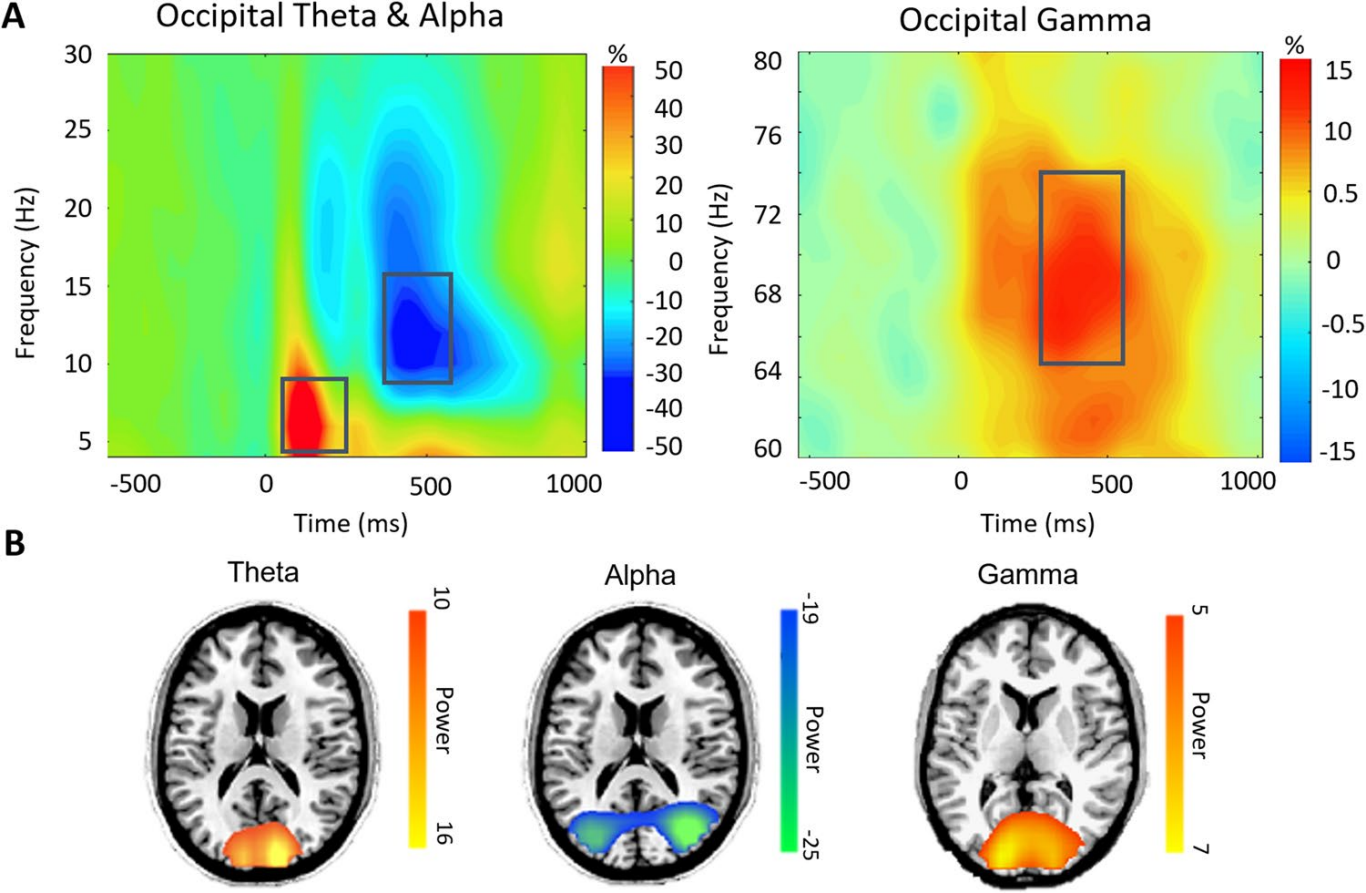
MEG sensor-level spectrograms and source images. (**A**) Sensor-level analysis revealed distinct oscillatory responses within the theta (4–8 Hz), alpha (8–16 Hz), and gamma (64–74 Hz) bands during visual processing in the occipital cortices. Each spectrogram reflects activity of a representative occipital MEG sensor that has been grand-averaged across all participants. The color scale legend shown on the right displays percent change from baseline (red indicating an increase in power relative to baseline and blue reflecting a decrease relative to baseline). (**B**) Significant sensor-level time–frequency components were imaged using a beamforming approach in each participant individually, and the resulting maps were grand-averaged across all participants per oscillatory response. The resulting functional maps reveal increases (i.e., synchronizations) in the theta and gamma range in the bilateral medial occipital cortices, as well as decreases in the strength (i.e., desynchronization) of alpha activity in more lateral occipital cortices. Color scale legends to the right of each image indicate average baseline-normalized power (pseudo-t) thresholds.

We identified visual-spatial oscillatory responses in the theta, alpha, and gamma ranges. Specifically, MEG sensors near the occipital cortices showed significant theta (4–8 Hz), alpha (8–16 Hz), and gamma (64–74 Hz) activity from 0 to 250 ms, 300 to 550 ms, and 250 to 550 ms, post-stimulus respectively (Fig. 1A). Beta (18–24 Hz) activity was also identified around participants’ reaction time in sensors above motor cortices, but these motor responses were beyond the scope of this study.

### Dynamic functional mapping

To identify the anatomical origin of the neural populations generating these sensor-level time–frequency responses, a beamforming approach was utilized. We used a pre-stimulus baseline period of equivalent duration and bandwidth to the target windows identified in the sensor level analysis to each window of interest (baselines: theta: − 300 to − 50 ms, alpha: − 300 to − 50 ms, gamma: −300 to 0 ms). The resulting functional maps were averaged across the entire sample in order to identify the specific location of visual-spatial neural oscillatory activity. Mapping of both the theta response and the gamma response revealed two (bilateral) clusters in medial occipital cortices near primary visual cortices. In contrast, alpha oscillatory activity showed two (bilateral) clusters in more lateral occipital cortices, near visual association areas (Fig. 1b).

### Reduced alpha power in alcohol use disorder

**Figure 2:**
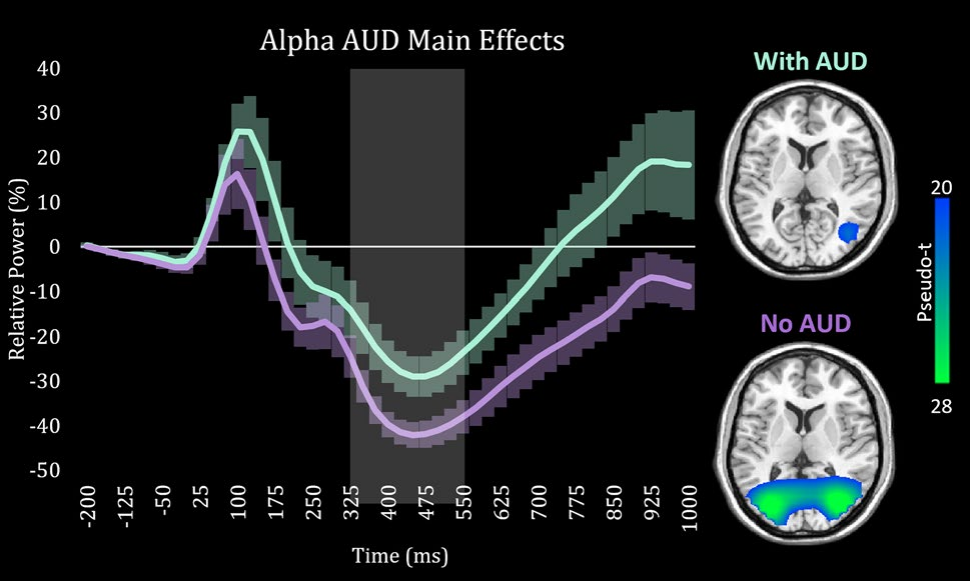
Voxel time series of alpha activity during visual-spatial processing. Grand averaged alpha source images revealed peak voxels in bilateral visual cortices. Time series were then extracted from these voxels in each participant, averaged across hemisphere, and were plotted with time (ms) on the x-axis and relative amplitude (%) on the y-axis. For display purposes, the time series has been averaged across those with AUD (teal) and those without AUD (purple). The area highlighted in gray (300 to 550 ms) reflects the time period that was used for the source analysis. Factorial ANCOVA showed a main effect of AUD, such that individuals diagnosed with AUD (teal) exhibited a significantly weaker alpha response compared to those without AUD (purple). Error bars indicate $\pm 1$ standard error of the mean. To the right, source images of the alpha response have been split according to AUD diagnosis with the color scale legend shown on the right. These images clearly show the same effect observed in the time series data. Note that these images are shown for display purposes only and statistical comparisons were made using the average over the highlighted active window from the extracted time series in an ANCOVA model.

Next, we computed voxel time series data from the peak voxels in each region, per participant and response, in order to probe for group differences. Theta, alpha, and gamma all showed symmetric bilateral clusters (one cluster per hemisphere), and since we did not have any laterality hypotheses, we averaged the time series across the hemispheres per oscillatory response in each participant prior to statistical analyses. This resulted in one time series per response, per participant. We then averaged the frequency power across the active window, and ran ANCOVAs to test for the effects of alcohol and cannabis use disorder. We utilized a 2 × 2 design to allow for the investigation of each main effect while controlling for the variance associated with all other variables. For example, the main effect of AUD therefore tests participants with AUD against those without AUD, controlling for CUD, sex, and age. For alpha oscillatory activity, ANCOVA revealed a significant main effect of AUD (*F*(1,40) = 8.12; *p* = 0.007; η^2^ = 0.163) but not CUD (*F*(1,40) = 3.11; *p* = 0.082), which indicated that those with AUD had blunted occipital alpha responses compared to those without AUD from 300 to 550 ms (Fig. 2). In contrast, our ANCOVA models showed no significant main effects of AUD or CUD on both theta and gamma band oscillations (all *ps* > 0.10), with the exception of an effect of reduced gamma activity in CUD (*F*(1,40) = 4.26; *p* = 0.046; η^2^ = 0.086). However this effect of CUD on gamma appeared to be driven by a participant with an outlier value (three standard deviations away from overall mean) as the effect was no longer significant upon excluding this data point (*F*(1,39) = 3.40; *p* = 0.072).

To ensure our results were not driven by other factors, we performed additional quality checks. We noted there were some participants with substance use disorders other than cannabis and alcohol (n = 6). To ensure our results were not driven by these participants, we added in a binary covariate indexing other substance use disorders, and our main effect of AUD remained significant in this model as well (*F*(1,38) = 5.73; *p* = 0.022; η^2^ = 0.131). We also examined the baseline alpha activity by extracting the average absolute alpha power from the baseline period, and neither AUD nor CUD showed significant effects (all *ps* > 0.10) using the same statistical model.

**Figure 3:**
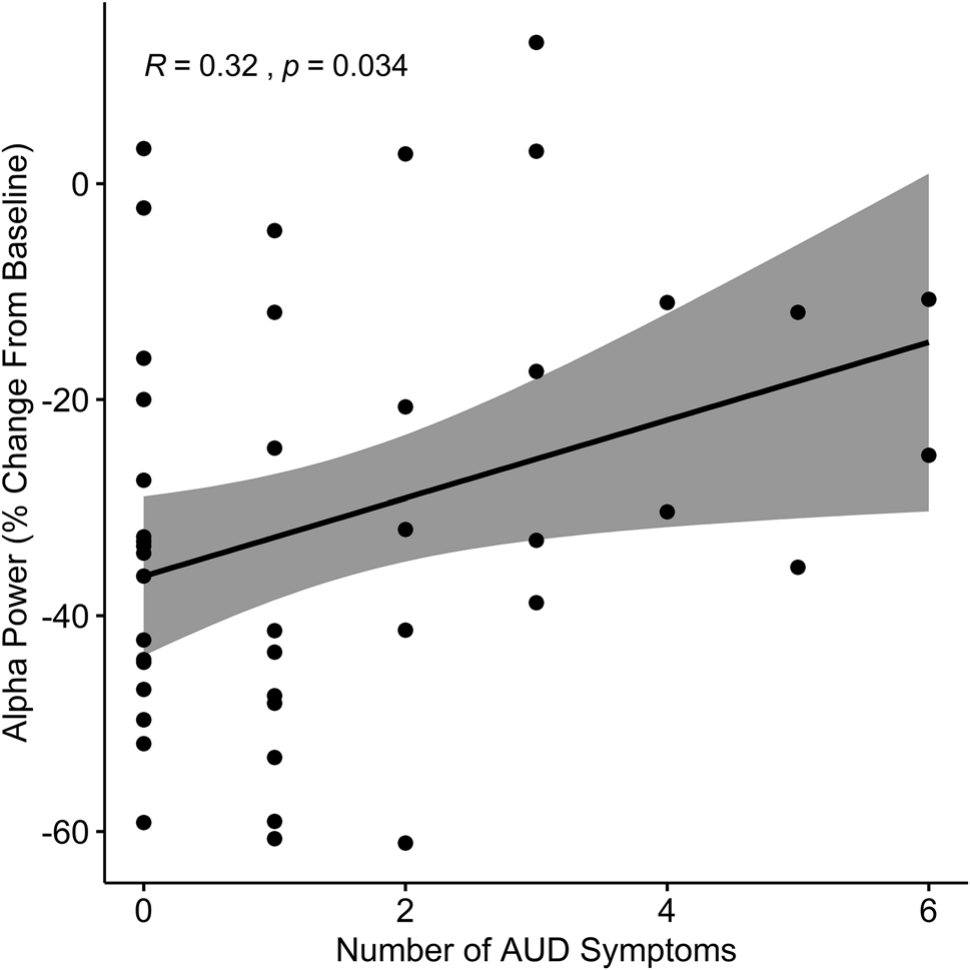
Spearman correlation between alpha power and number of AUD symptoms. Post-hoc testing of alpha power showed a significant correlation between number of AUD symptoms and alpha power such that participants that had more severe AUD exhibited weaker alpha responses. This relationship remained significant in a larger regression model that included number of CUD symptoms, and metrics of cannabis and alcohol consumption, showing that decreased alpha responses are associated with more severe AUD, above and beyond amount of alcohol consumption and cannabis consumption/use disorder. Note that more negative alpha power indicates a stronger alpha response (larger change from baseline). Shaded area represents 95% confidence interval.

Finally, to examine the substance-related factors driving the alpha effects, we ran several additional tests. First, we ran a bivariate spearman correlation of alpha oscillatory power with number of AUD symptoms. This showed a significant correlation such that a greater number of AUD symptoms was associated with a weaker alpha response (*r*(42) = 0.32, *p* = 0.034). To determine whether this relationship was driven by the amount of substance use, we next performed multiple regression with AUD and CUD symptoms, and AUDIT/CUDIT-C scores as predictors on alpha activity. This showed that the number of AUD symptoms remained the only significant predictor above and beyond the other variables (t(39) = 5.53; *p* = 0.024; Fig. 3).

## Discussion

Our study focused on the independent effects of AUD and CUD in a sample of 45 adults reporting current use of both substances. We found that in adults using both cannabis and alcohol, those with AUD had significantly weaker alpha responses during visual-spatial processing within the lateral occipital cortices. Additionally, reduced alpha responses were related to increased AUD symptoms, indicating that reduced alpha power scales with more severe AUD. In contrast, no effects of CUD were identified in this sample. This study is the first to look at oscillatory alterations during visual processing in the context of substance use disorders.

Our findings are particularly interesting given that our study was specifically designed to examine the neural alterations related to addiction amongst a population of alcohol and cannabis users. This is in contrast to studies that examine alcohol/cannabis use compared to nonuser controls. By using a control group that consisted of substance users, this study effectively probed the neural activity related to pathologic use, as opposed to any use. Thus, our study design should have been particularly sensitive to addiction-related aberrations, which may drive compulsive substance use. Identification of such networks is crucial in understanding substance use disorders and may be helpful in guiding targeted therapies in the future. Alternatively however, our findings could have also reflected circuits and oscillatory responses that are altered with substantial use of the substance, since use that is more chronic often co-occurs with more severe use disorder. This would have suggested frequency-specific alterations related to consumption of large quantities of the substance. To address this, we performed follow-up regression analyses that included number of use disorder symptoms, as well as measures of the frequency of alcohol and cannabis use to help decipher whether our findings were associated with addiction-related circuitry, or with amount of substance use. This is notable because, in the SCID interview, use disorder symptoms, and respective use disorder diagnosis, do not inquire about the amount or regularity of use. Ultimately, this showed that reduced alpha power was significantly associated with increased number of AUD symptoms, above and beyond the amount of alcohol and cannabis use, and the number of CUD symptoms. This statistically narrows our neural findings toward being specifically related to AUD symptom severity, further supporting that the alpha reductions we observed were addiction-related.

Importantly, this regression analysis also clarifies our results with respect to our previous study which identified reduced alpha responses in participants with heavy alcohol use compared to a participant group with minimal to no alcohol use^34^. The current study also found a reduction in alpha responses, but in a comparison of participants with AUD against a non-use disorder comparison. Therefore, given this same alteration is found in both heavy alcohol users and those with AUD, our regression analysis focused on teasing out whether the amount of alcohol use or use disorder symptoms better explain these changes. Ultimately, our results clarify that such a reduction in alpha responses are in fact more closely associated with use disorder severity, rather than quantity of use. This is an important and novel finding which highlights the importance of oscillatory activity in AUD. Further study however is needed to confirm these findings in a larger sample.

In the context of other literature, it is intuitive that the blunted alpha activity identified in those with AUD may index alterations in alcohol addiction-related activity. Visual alpha activity is consistently related to attention processing in visual-spatial paradigms and has been shown to be modulated by frontoparietal networks^36,37^. Attention and frontoparietal networks have been shown to be specifically implicated in AUD^38^ and a reduction in alpha responsiveness may index dysfunction in these networks. In contrast, primary visual theta and gamma have been associated with basic visual processing^29,39^, which would most likely remain intact. Therefore, future studies should further examine whether these alpha differences are related to attentional processing, as the current study did not directly manipulate attention. Broadly, our findings also fit into a growing literature showing altered oscillatory processing related to AUD during a host of cognitive tasks^40–42^, which together indicate that neural oscillatory activity may serve as a useful marker for measuring brain dysfunction explicitly related to AUD.

While chronic cannabis use is known to have detrimental effects on neural circuitry^43,44^, and the networks underlying CUD are beginning to be identified, we did not identify any effects of CUD on visual oscillatory activity in this sample. Cannabinoid-1 receptors (CB1Rs) control the release of neurotransmitters such as GABA and glutamate when activated by cannabinoids, whether that being naturally occurring or from marijuana. Indeed, disruptions in oscillatory activity have been identified with acute cannabis use, and GABAergic interneurons have been deliberated as the cause of such oscillatory disruption^9^. However, our study did not assess oscillatory activity in the context of short-term drug effects, and such acute effects of cannabis are unlikely to generalize to our sample of chronic users who had not recently consumed such substances. Still other studies have identified alterations in chronic cannabis users in oscillatory activity related to attentional processing^45,46^. Compared to these studies however, the current study utilized a paradigm that did not utilize an attentional comparison. Additionally, our study was designed to investigate the effects of use disorder, and therefore our test of the main effect of CUD effectively compared cannabis users without use disorder to cannabis users with CUD. Thus, our lack of CUD findings may simply indicate that the addiction circuitry related to CUD may not involve these particular visuo-spatial processing responses. Although speculative, the independence of our AUD effect may indicate specificity that could be useful in the development of AUD biomarkers. That is, although AUD and CUD are addictive disorders that show high rates of comorbidity with each other, our findings may reflect underlying pathology that makes these disorders distinct rather than the potentially overlapping pathology that makes addictive disorders highly comorbid.

Before closing, it is important to note the limitations of this study. First, we note that our sample size is relatively small. To ensure maximal power in this sample, we therefore focused on the main effects of AUD and CUD, which utilize n_AUD_ = 17 versus n_non-AUD_ = 28, and n_CUD_ = 26 versus n_non-CUD_ = 19 comparisons. Conducting the comparisons in this way ensured that our samples were of moderate size for detecting effects in the context of MEG. Further studies need to be conducted with a larger sample size to replicate our findings, as well as investigate potential interactive effects. Additionally, to be fully inclusive in our sample, we included a number of participants (n = 6) who had other co-occurring SUDs. These other use disorders varied from participant to participant and no conclusions could be drawn about the independent or combined effects of these additional use disorders due to insufficient statistical power. Instead, as a post-hoc analysis, we tested whether our effect remained after a binary covariate indexing other SUDs was used. A similar covariate approach was made to account for the fact that participants with AUD were much more likely to be male than female. While our results remained significant, this approach is imperfect, and additional studies are warranted to determine the impact of other SUDs and effects of biological sex. Future studies should aim to recruit more females with AUD as, although males have a higher rate of AUD than females, this gender gap appears to be closing^47^. Third, our study utilized AUDIT/CUDIT measures to derive additional information on amount/frequency of use, but was limited in details such as concurrent versus simultaneous use, time of last use, and tobacco use, and future study is needed to assess these variables. Finally, our task probed visual-spatial processing, which was largely limited to neural activity in the occipital cortices. Further studies are needed to understand how these changes affect and interact with more frontally-mediated executive function deficits that can occur in substance use disorders.

In conclusion, we identified use disorder specific effects on neural oscillatory activity during a visual-spatial processing task. Amongst a sample of cannabis and alcohol users, reductions in the occipital alpha response were seen in individuals with AUD, and this scaled with increasing severity of AUD. Given that our model included main effects of both AUD and CUD, our AUD findings were statistically independent of CUD, which could reflect the specificity of alpha reductions in AUD. Overall, our findings suggest alpha oscillatory activity may play an integral role in the neural networks related principally to alcohol addiction. With further study, this neural activity could be used not only to further understand the circuitry of AUD, but may even be useful as a phenotypic marker of AUD.

## Methods and materials

### Participants

Forty-five participants (age range: 19–56 years) that reported current use of both alcohol and cannabis were recruited from the community and enrolled in the study. The structured clinical interview for DSM-V (SCID-5-RV) was administered to every participant in order to ensure a systematic evaluation and accurate diagnosis of substance use disorders^48^. Interviewers were trained and overseen by a clinical psychologist, and all participants completed the interview process. Participants’ number of symptoms for current alcohol use disorder (AUD) and cannabis use disorder (CUD) were utilized to categorize participants into positive/negative AUD/CUD. Participants also completed the Alcohol Use Disorder Identification Test (AUDIT^49^) and Cannabis Use Disorder Identification Test (CUDIT-R^50^), underwent urine drug screens to confirm self-reported recent substance use, and were instructed not to use substances immediately prior to participation in the study. Exclusionary criteria included any diagnosed psychiatric disorder other than substance use disorder, current use of psychiatric medication, any other chronic medical illness affecting CNS function, pregnancy, and presence of any ferrous metal implant (orthodontures, cardiac pacemaker, surgical implants, etc.) which may interfere with the MEG/MRI data acquisition. The institutional review board at the University of Nebraska Medical Center approved this investigation and each participant provided written informed consent prior to participation in the study. All methods were carried out in accordance with relevant guidelines and regulations.

### Visual-spatial processing task

**Figure 4:**
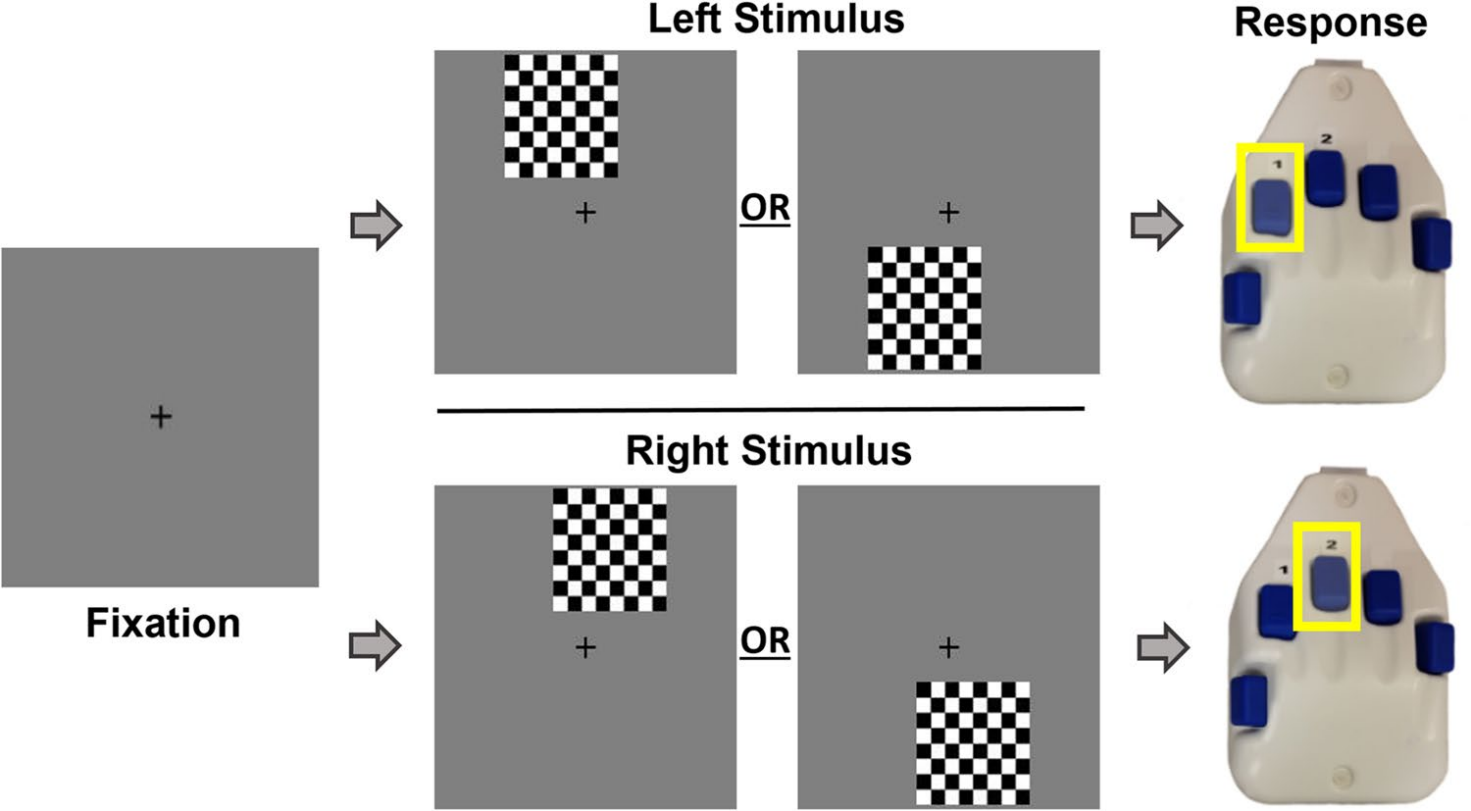
Visual-spatial processing task. Every trial began with a fixation period lasting 1900 to 2100 ms, which was then followed by the stimulus grid (duration = 800 ms). The stimulus grid was presented in one of four locations; off-center by 75% to either the right or the left of the fixation crosshair in the top or lower quadrant. Participants were instructed to use the response pad to indicate whether the grid was presented to the left (index finger) or right (middle finger).

Participants completed a visual-spatial processing task while undergoing MEG recording. This task has been used to elicit multiple spectrally-specific patterns of neural activity^24,34^. While participants were seated in a non-magnetic chair in a magnetically-shielded room, they were instructed to focus on a centrally located fixation crosshair for about 2000 ms (variable inter-stimulus range of 1900–2100 ms). An 8 × 8 stimulus grid was then shown for 800 ms in one of four positions relative to the fixation point: above and to the right, above left, below right, or below left (Fig. 4). A photodiode was used to measure the trigger to stimulus delay, and this delay was accounted for throughout the analysis. Participants were instructed to respond as fast as they could using their right hand placed on a five finger button pad, indicating whether the grid was presented to the left or the right of the fixation crosshair (Fig. 4). Each individual underwent 240 trials of this task and only the correct trials were analyzed. To account for spurious reaction times, we performed standard data trimming procedures for each participant by excluding reaction times 3 SD above or below each participant’s mean prior to averaging.

### MEG recording

MEG data were recorded in a one-layer magnetically-shielded room with active shielding engaged to compensate for environmental noise. Neuromagnetic responses were sampled at 1 kHz using an Elekta MEG system (Helsinki, Finland) equipped with 102 magnetometers and 204 planar gradiometers. All analyses focused on the data from the 204 planar gradiometers. Participants were monitored during MEG acquisition by real-time audio–video feeds from inside the room. Each participant’s data was individually corrected for head movement using four position indicator coils attached to the participant’s head, which were continuously measured throughout recordings. The data were also subjected to noise reduction using the temporally-extended signal space separation method^51,52^.

### MEG data processing and sensor level statistics

Noise-reduced MEG data underwent standard data preprocessing procedures using Brain Electrical Source Analysis software (BESA version 7.0). This included visual inspection for large artifacts, such as cardiac and blink artifacts, which were corrected for using signal-space projection (SSP^53,54^, and coregistration of each participant’s MEG data with their structural T1-weighted MRI data. The MEG data were then split into 2000 ms epochs (− 500 to 1500), with 0.0 s defined as stimulus grid onset. Epochs containing artifacts were then rejected using a fixed threshold method based on participant-specific amplitude and gradient thresholds. In addition, we confirmed that the number of correct trials analyzed did not significantly differ by AUD and CUD, including covarying for sex and age (AUD: *F*(1,40) = 2.23, *p* = 0.143; CUD: *F*(1,40) = 0.31, *p* = 0.581), which helps ensure that the signal-to-noise ratio was comparable between groups.

For each sensor, artifact-free epochs were transformed into the time–frequency domain using complex demodulation^55,56^. This involves filtering the complex signal into frequency bands of a predetermined width and range, and calculating the power within each band across each successive temporal window. The resulting spectral power estimations per sensor were then averaged across trials and normalized by calculating the percent change in power relative to the baseline time period (− 400 to 0 ms) per frequency bin. Time–frequency windows of interest were then identified using paired-samples t-tests against baseline on each data point in the sensor-level spectrograms across all participants’ gradiometers (*p* < 0.05), and then correcting for multiple comparisons using nonparametric cluster-based permutation testing^57,58^.

### MEG source imaging and voxel time-series

To image cortical activity in the statistically-determined time–frequency windows of interest, we utilized the dynamic imaging of coherent sources (DICS) beamformer to calculate voxel-wise source power across the entire brain volume for each participant^59,60^. The resulting images were normalized using a pre-stimulus baseline period of equal duration and bandwidth to the time–frequency window of interest. Such source images are commonly referred to as pseudo-t maps, with the unit (pseudo-t) reflecting noise-normalized power differences (i.e., task vs. baseline) per voxel^61^. Source images were computed at 4.0 × 4.0 × 4.0 mm resolution and were transformed into standardized space. The resulting images were averaged across all participants (collapsed across group) to identify voxels with the strongest responses per time–frequency bin of interest.

For each peak voxel in the grand-averaged functional maps, voxel time series were computed for the specific coordinate per participant. Briefly, in each participant, we applied the individual sensor-weighting matrix to the preprocessed signal vector, which yielded a time series for each source vector centered in the voxel of interest. The vector sum of the two orientations was then computed and normalized using the baseline period from the source analysis, ultimately yielding one time series per voxel per participant. For each person, the average over the respective time–frequency windows of interest for theta, alpha, and gamma frequency were computed using these time series, and these values were used in the statistical analysis.

### Statistical analyses

To test for the effects of alcohol use disorder (AUD) and cannabis use disorder (CUD), we utilized 2 × 2 factorial ANCOVA models, with AUD and CUD as predictor variables, each with two levels (use disorder versus no use disorder). Additionally, to account for demographic differences, we added age and sex as covariates into the models. This design was utilized on behavioral performance (accuracy and reaction time), as well as neural activity in each time–frequency window of interest to interrogate the independent main effects of AUD and CUD. Our design was specifically aimed at testing the independent main effects of AUD and CUD to maximize statistical power. That is, the main effect of AUD compares all participants with AUD against all participants without AUD. Because the effects of AUD and CUD are examined in the same model, the effect of the other use disorder is controlled for, effectively controlling for comorbid use disorders. Interaction terms were not included due to the relatively small sample size of the subgroups.

Post-hoc testing was then performed to further understand the nature of our effects. First, while only a few participants exhibited a substance use disorder other than AUD and CUD, we added a binary substance use disorder (SUD) covariate into the ANCOVA models to investigate whether any effects were driven by other SUDs. Then we interrogated whether our effects were driven by AUD/CUD severity, versus the amount/frequency of alcohol/cannabis use. To index use disorder severity, we used number of use disorder symptoms for AUD and CUD from the SCID interview. To index amount/frequency of use, we utilized scores from the first three questions of the AUDIT and CUDIT questionnaires, which is often known as the AUDIT-C/CUDIT-C^62^. These questions inquire about frequency of use (Q1), amount of use (Q2), and binge (Q3) use, which importantly is not assessed in the SCID interview. That is, use disorder symptoms and diagnosis do not take into account amount or frequency of use. Therefore, we used these four variables (AUD/CUD number of symptoms based on the SCID and A/CUDIT-C score) in a regression to determine whether our effects were related to amount of alcohol/cannabis use or alcohol/cannabis addiction symptomatology.
